# Supplementary material for: The Use and Effectiveness of Selected Alternative Markers for Insulin Sensitivity and Secretion Compared with Gold Standard Markers in Dietary Intervention Studies in Individuals without Diabetes: Results of a Systematic Review
Source: Nutrients. 2022 May 12;14(10):2036. doi: 10.3390/nu14102036 (PMC9143618; doi:10.3390/nu14102036)
Supplement: Supplementary file 1 [file nutrients-14-02036-s001.zip › Supplemental Material Figure S1.pdf]

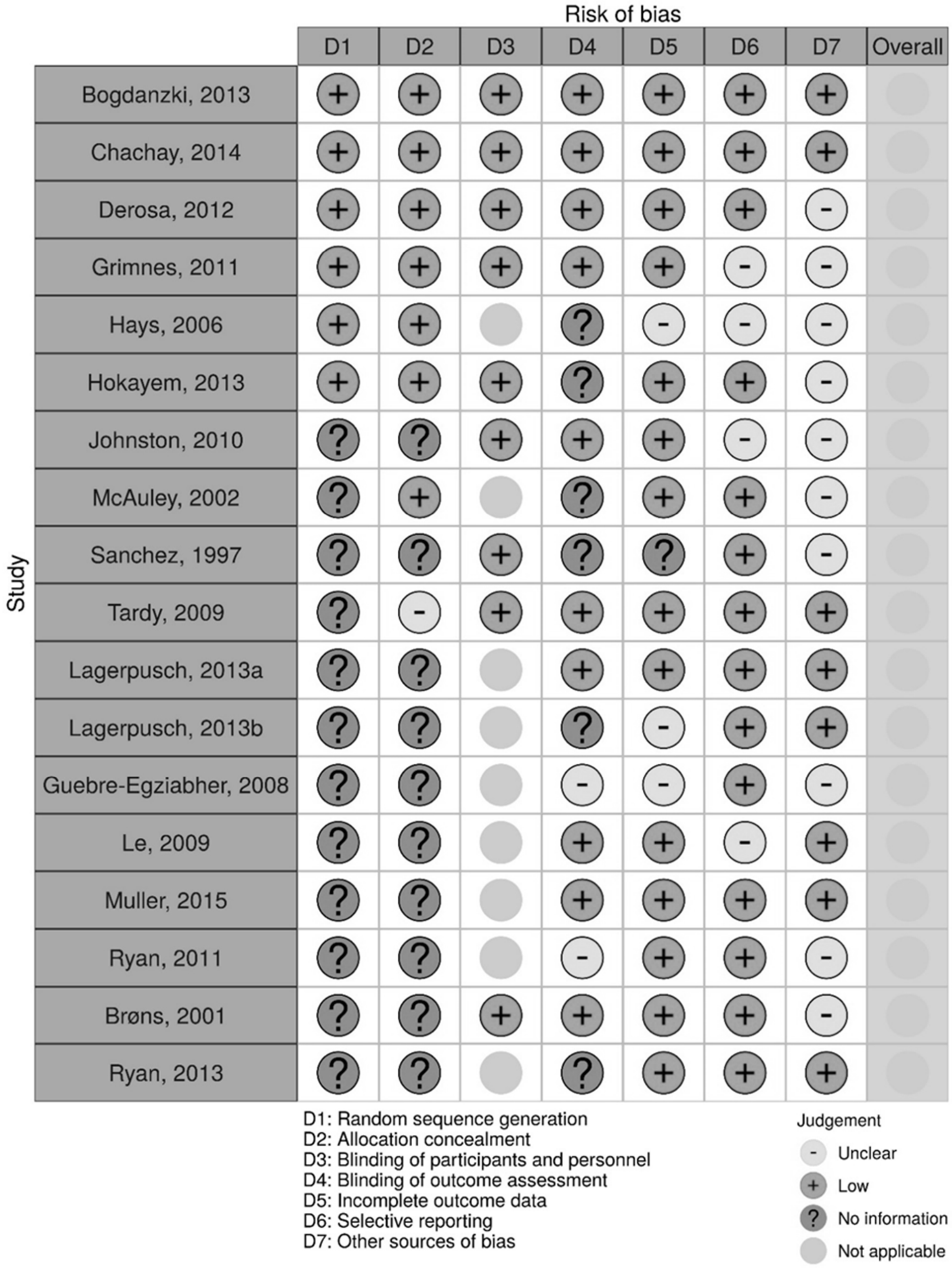

Figure S1. Risk of bias assessment, Euglycaemic hyperinsulinaemic clamp.
