# Supplementary material for: The Use and Effectiveness of Selected Alternative Markers for Insulin Sensitivity and Secretion Compared with Gold Standard Markers in Dietary Intervention Studies in Individuals without Diabetes: Results of a Systematic Review
Source: Nutrients. 2022 May 12;14(10):2036. doi: 10.3390/nu14102036 (PMC9143618; doi:10.3390/nu14102036)
Supplement: Supplementary file 1 [file nutrients-14-02036-s001.zip › Supplemental Material Table S1.pdf]

Table S1. Results of the Euglycaemic-hyperinsulinaemic clamp trials and the surrogate markers.

	Euglycaemic-hyperinsulinaemic clamp		AUC OGTT		HOMA-IR		Fasting insulin	
**Study**	**Baseline**	**End of intervention**	**Baseline**	**End of intervention**	**Baseline**	**End of intervention**	**Baseline**	**End of intervention**
**Bogdanzki, 2013**	L-arginine=3.2± 1.8 mg/kg/min (SE)	L-arginine=4.3± 2.1 mg/kg/min (SE)**, PC=34.38%					L-arginine=9.2±13.9 µUI/MI (SE)	L-arginine=25.5±12.7 (SE)** PC= -12.67%
**Chachay, 2014**	Resveratrol=3.5±2.7 mg/kgLBW/min (SD)	Resveratrol=3.2±2.8 mg/kgLBW/min (SD), PC= -8.57%			Resveratrol=2.8±1.8 (SD)	Resveratrol=3.6±1.9 (SD), PC= 28.57%		
**Derosa, 2012**	n-3 PUFAs group=5.58±0.81 µmol/min/kg (SD)	n-3 PUFAs group=7.47±1.98 µmol/min/kg (SD)**, PC= 33.87%			n-3 PUFAs= 2.08± 1.09 (SD)	n-3 PUFAs= 1.81±0.92 (SD), PC= -12.98%		
**Grimnes, 2011**	Vit D=33.9±15.5 µmol min^{-1} kg^{-1} (SD)	Vit D= 38.5±15.7 µmol min^{-1} kg^{-1} (SD), PC= 13.57%			Vit D=1.83±0.59 (SD)	Vit D=2.24±0.64 (SD), PC= 22.4%		
**Hays, 2006**	Men EX+ Diet= 3.02±0.17 mg kg^{-1}min^{-1} (SEM)	Men EX+ Diet= 3.64±0.27 mg kg^{-1}min^{-1} (SEM)**, PC= 20.53%	Men EX+ Diet= 12.1±1.1 µU/ml (SEM)	Men EX+ Diet= 15.6±3.8 µU/ml (SEM), PC= 29.92%	Men EX+ Diet= 2.9±0.3 (SE)	Men EX+ Diet= 3.7±0.8 (SE), PC= 27.58%		
**Hokayem, 2013**	Grape PP=10.9±1.0 mg kg $^{-1}$min^{-1} (SEM)	Post 8 weeks PP=11.4±0.7 mg kg $^{-1}$min^{-1} (SEM), PC= 4.59% After fructose supplementation PP=11.4 ±1.1 mg kg $^{-1}$min^{-1} (SEM), PC= 4.59%					PP=4.4±0.4 mU/L (SE)	PP: After 8 weeks=4.4±0.3 mU/L (SE), PC= O After fructose=4.4±0.4 mU/L (SE), PC= 0
**Johnston, 2010**	Resistant starch (RS)=5.8±7.2 min kg pmol (SEM)	RS=6.7±8.1 min kg pmol (SEM)** PC= 15.52%			RS= 68.5±4.98 (SE)	RS= 80.2±12.7 (SE), PC= 17.08%		
**McAuley, 2002**	Moderate exercise(M)=4.6±1.3 mg kg^{-1}min^{-1} (SD), Intensive exercise (I)=5.1±1.6 mg kg^{-1}min^{-1} (SD)	M=5.4±1.5 mg kg^{-1}min^{-1} (SD), PC= 17.39% I= 6.3±2.1 mg kg^{-1}min^{-1} (SD)**, PC= 23.53%					M=17.5±11.7 mIU/l (SD) I=17.0±7.9 mIU/l (SD)	M=16.5±11.1 mIU/l (SD), PC= -5.71% I=12.9±5.4 mIU/l (SD), PC= -24.12%
**Sanchez, 1997**	Calcium=2.89±0.77 mg kg^{-1}min^{-1} (SE)	Calcium= 4±0.95 mg kg^{-1}min^{-1} (SE)**, PC= 38.41%					Calcium=71.8±5.9 pmol/l (SE)	Calcium=64.6±6.2 pmol/l (SE)**, PC= -10.03%
**Tardy, 2009**	Low-Trans Fatty Acids=5.64±2.49 mg kg body wt^{-1}min^{-1} (SD), Ruminant-TFA=5.57±1.37 mg kg body wt^{-1}min^{-1} (SD),	Low-TFA=5.64±2.01 mg kg body wt^{-1}min^{-1} (SD), PC= O R-TFA=5.71±1.65 mg kg body wt^{-1}min^{-1} (SD), PC= 2.51% IP-TFA=5.36±2.26 mg kg body wt^{-1}min^{-1} (SD), PC= 3.28%			Low-TFA=2.25±1.35 (SD), R-TFA=3.26±3.08 (SD), IP-TFA=2.95±2.02 (SD)	Low-TFA=2.36±1.31 (SD), PC= 4.89% R-TFA=2.79±2.20 (SD), PC= -14.42% IP-TFA=3.09±2.32 (SD), PC= 4.75%		

	Industrial Product Source-TFA=5.19±2.47 mg kg body wt^{-1}min^{-1} (SD)							
**Lagerpusch, 2013a**	Low-glycaemic index: 50%CHO=9.37±2.15 mg/(kg x min) (SD), 65%CHO=8.66±3.36 mg/(kg x min) (SD)	Low-glycaemic index: 50%CHO=8.49±2.44 mg/(kg x min) (SD), PC= -9.39% 65%CHO=8.19±1.55 mg/(kg x min) (SD), PC= -5.43%	Low-glycaemic index: 50%CHO= 90.9±41.1 AUC mU/(L x 3h) (SD), 65%CHO= 128±56.8 mU/(L x 3h) (SD)	Low-glycaemic index: 50%CHO= 86.1±46.3 mU/(L x 3h) (SD), PC= -5.28% 65%CHO= 168±151 mU/(L x 3h) (SD), PC= 31.25%	Low-glycaemic index: 50%CHO=1.14±0.54 (SD), 65%CHO=2.19±1.72 (SD)	Low-glycaemic index: 50%CHO=0.98±0.28 (SD), PC= -14.04% 65%CHO=1.89±1.08 (SD), PC= -13.7%		
**Lagerpusch, 2013b**	Low-Glycaemic index (LGI) group=11.23±2.54(mg/kg FFM per min) (SD)	LGI group=9.92±2.35 (mg/kg FFM per min) (SD), PC= -11.67%	LGI=90.84±40.98 AUC mmol/l per 3 h(SD)	LGI=86.24±46.17 AUC mmol/l per 3 h (SD), PC= 4.74%	LGI=1.14±0.54 (SD)	LGI=0.98±0.27 (SD), PC= -14.03%		
**Guebre-Egziabher, 2008**	Baseline=9.54±3.34 mg/kg/min (SD)	After 10 weeks of n-3 polyunsaturated fatty acids (PUFA)= 9.96±2.27 mg/kg/min (SD), PC= 4.4%					Baseline=42.3±14.4 pmol/l (SD)	After 10 weeks=37.4±10.5 pmol/l (SD), PC= -11.58%
**Le, 2009**	Isocaloric=6.2 ±0.3 mg kg^{-1} min^{-1} (SE),	High Fructose=4.8±0.2 mg kg^{-1} min^{-1} (SE)**, PC= 22.58%					Isocaloric=49±2 pmol/l (SE)	High Fructose=60±3 pmol/l (SE)**, PC= 22.45%
**Muller, 2015**	Basal=8.89±2.8569 mg kg^{-1}min^{-1} (SD)	14 d of refeeding=8.71±2.182 mg kg^{-1}min^{-1} (SD), PC= -2.02%					Basal=7.94±4.147 mU/L (SD)	14d refeeding=8.20±4.355 mU/L (SD), PC= 3.27%
**Ryan, 2011**	Low fat-high carbohydrate diet (LF/HCD)=2.2±0.8 mg/kg^{-1}/min^{-1} (SD)	Mediterranean Diet (MD)=4.2±2.8 mg/kg^{-1}/min^{-1} (SD)**, PC= 90.90%			LF/HCD =4.7±1.6 (SD)	MD=3±1.4 (SD)**, PC= -36.17%		
**Brøns, 2001**	Taurine= 10.2±4.5 (mg glu/kg FFM/min) (SD)	Placebo=9.7±0.5 (mg glu/kg FFM/min) (SD), PC= 4.9%			Taurine=1.99±1.15 (SD)	Placebo=1.82±0.99, PC= 8.54%		
**Ryan, 2013**	AEX (aerobic exercise) +CR (calorie restriction): NGT (normal)=69.1±2.9 (μmol kg FFM^{-1}min^{-1}) (SE), IGT (impaired glucose tolerant)=56±5.1 (μmol kg FFM^{-1}min^{-1}) (SE)	AEX+CR: NGT=77.4±2.9 (μmol kg FFM^{-1}min^{-1}) (SE), PC= 12.01% IGT=62.2±4.0 (μmol kg FFM^{-1}min^{-1}) (SE), PC= 11.07%					AEX+CR: NGT=67±3 pmol/l (SE), IGT=109±15 pmol/l (SE)	AEX+CR: NGT=53±3 pmol/l (SE), PC= -20.9% IGT=85±9 pmol/l (SE), PC= -22.02%

**$p \leq 0.05$, SD= Standard deviation, SE= Standard error of the mean. PC= Proportional change between baseline and intervention %, HOMA-IR=Homeostatic Model Assessment for Insulin Resistance, AUC OGTT= Area Under the Curve-Oral Glucose Tolerance Test.
