# Supplementary material for: The Use and Effectiveness of Selected Alternative Markers for Insulin Sensitivity and Secretion Compared with Gold Standard Markers in Dietary Intervention Studies in Individuals without Diabetes: Results of a Systematic Review
Source: Nutrients. 2022 May 12;14(10):2036. doi: 10.3390/nu14102036 (PMC9143618; doi:10.3390/nu14102036)
Supplement: Supplementary file 1 [file nutrients-14-02036-s001.zip › Supplemental Material Table S2.pdf]

Table S2. Results of the Intravenous Glucose Tolerance Test trials and the surrogate markers.

	IVGTT (S_i)		AUC OGTT		HOMA-IR		Fasting insulin	
**Study**	**Baseline**	**End of intervention**	**Baseline**	**End of intervention**	**Baseline**	**End of intervention**	**Baseline**	**End of intervention**
**Alemzadeh, 1998**	Diazoxide= 0.77±0.18 (min^{-1} uU^{-1} mL x 10^{4}) (SE)	Diazoxide= 1.07±0.21 (min^{-1} uU^{-1} mL x 10^{4}) (SE), PC= 38.96%					Diazoxide= 168±14 pmol/l (SE)	Diazoxide=108±13 pmol/l (SE)**, PC= -35.71%
**Osterberg, 2015**	VSL#3 probiotic=5.9±1.3 [(mU L^{-1}) min^{-1}] (SE)	VSL#3 probiotic=5.5±0.9 [(mU L^{-1}) min^{-1}] (SE), PC= -6.78%					VSL#3 probiotic= 6.1±1.5 mIU ml^{-1} (SE)	VSL#3 probiotic= 5.9±1.4 mIU ml^{-1} (SE), PC= -3.28%
**Jans, 2012**	High Monounsaturated Fatty Acid (HMUFA)=3.04±0.28 (Mu/L^{-1}min^{-1}10^{-4}) (SE),<br>Low Fat High Complexed Carbohydrate (LFHCC)=3.02±0.66 (Mu/L^{-1}min^{-1}10^{-4}) (SE),<br>Low Fat High Complexed Carbohydrate n-3 PUFA (LFHCC n-3)=2.71±0.30 (Mu/L^{-1}min^{-1}10^{-4}) (SE),<br>High Saturated Fatty Acid (HSFA)=3.47±0.73 (Mu/L^{-1}min^{-1}10^{-4}) (SE)	HMUFA=2.69±0.30 (Mu/L^{-1}min^{-1}10^{-4}) (SE), PC= -11.51%<br>LFHCC=3.21±0.43 (Mu/L^{-1}min^{-1}10^{-4}) (SE), PC= 6.29%<br>LFHCCn-3=2.99±0.31 (Mu/L^{-1}min^{-1}10^{-4}) (SE), PC= 10.33%<br>HSFA=3.25±0.41 (Mu/L^{-1}min^{-1}10^{-4}) (SE), PC= -6.34%			HMUFA=2.26±0.21 (SE),<br>LFHCC=2.88±0.57 (SE),<br>LFHCCn-3=2.20±0.45 (SE),<br>HSFA=2.18±0.27 (SE)	HMUFA=2.27±0.30 (SE), PC= 0.44%<br>LFHCC=2.70± 0.70 (SE), PC= -6.25%<br>LFHCCn-3=2.03±0.26 (SE), PC= -7.73%<br>HSFA=2.40±0.37 (SE), PC= 10.09%		
**Ard, 2004 W**	Group A (advice only)=2.2± 1.13 (mg kg^{-1}min^{-1}) (SD),<br>Group B (established protocol) = 2.32± 0.65 (mg kg^{-1}min^{-1}) (SD),<br>Group C (established protocol + DASH diet)=1.96± 0.94 (mg kg^{-1}min^{-1}) (SD)	Group A=2.2± 1.12 (mg kg^{-1}min^{-1}) (SD), PC= 0%<br>Group B=2.97±1.74 (mg kg^{-1}min^{-1}) (SD), PC= 28.02%<br>Group C=2.95±1.62 (mg kg^{-1}min^{-1}) (SD)**, PC= 50.51%					Group A= 16.65±9.05 µU/ml (SD),<br>Group B=14.14±5.23 µU/ml (SD)<br>Group C=15.65± 7.99 µU/ml (SD)	Group A=18.41±12.55 µU/ml (SD), PC= 10.57%<br>Group B= 10 ± 3.35 µU/ml (SD)**, PC= -30.56%<br>Group C=13.76±8.84 µU/ml (SD), PC= -12.08%
**%Davy, 2002**	Oat=1.8±0.3 (×10^{-4}/min^{-1}/µU^{-1}/ml^{-1}) (SE)	Oat=1.8±0.3 (×10^{-4}/min^{-1}/µU^{-1}/ml^{-1}) (SE), PC= 0%					Oat= 93.8±15.4 pmol/L (SE)	Oat= 88.4±12.5 pmol/L (SE), PC= -5.76%
**Kolehmainen, 2012**	Bilberries (BB)=2.31±1.00 ((mU/L)-1 x min-1) (SD)	BB=2.42±1.32 ((mU/L)-1 x min-1) (SD), PC= 4.76%	BB=14.5±10.2 mU/l (SD)	BB=14.4±8.1 mU/l (SD), PC= -0.68%			BB=14.5±10.2 mU/l (SD)	BB=14.4±8.1 mU/l (SD), PC= -0.69%
**Larson-Meyer, 2006**	Calorie Restriction (CR)=3.3±0.5 (10^{-4} mU 1^{-1} min^{-1}) (SE),<br>Calorie Restriction + Exercise (CREX)=3.4±0.4 (10^{-4} mU 1^{-1} min^{-1}) (SE),<br>Low-calorie diet (LCD)=3.1±0.6 (10^{-4} mU 1^{-1} min^{-1}) (SE),	CR=4.2±1.0 (10^{-4} mU 1^{-1} min^{-1}) (SE)**, PC=27.27%<br>CREX=5.3±0.8 (10^{-4} mU 1^{-1} min^{-1}) (SE)**, 55.88%<br>LCD=4.7±0.9 (10^{-4} mU 1^{-1} min^{-1}) (SE)**, PC=51.61%					CR= 9.4±1.5µu/ml (SE)<br>CREX= 9.8±1.0 µu/ml (SE)<br>LCD= 11.0±0.9 µu/ml (SE)	CR= 6.6±0.9 µu/ml (SE)**, PC= -29.79%<br>CREX= 7.0±0.7 µu/ml (SE)**, PC= -28.57%<br>LCD= 9.4±1.3 µu/ml (SE)**, PC= -14.55%

**Tierney, 2011**	HMUFA (high-monounsaturated fatty acid)=2.89±0.17 (mul^{-1})$^{-1}$min^{-1} (SE),<br>LFHCC (low-fat, high complex carbohydrate)=2.75±0.18 (mul^{-1})$^{-1}$min^{-1} (SE),<br>LFHCC n-3=2.58±0.19 (mul^{-1})$^{-1}$min^{-1} (SE),<br>HSFA(high-saturated fatty acid)=3.09±0.19 (mul^{-1})$^{-1}$min^{-1} (SE)	HMUFA=2.89±0.16 (mul^{-1})$^{-1}$min^{-1} (SE), PC= 0%<br>LFHCC=2.79±0.17 (mul^{-1})$^{-1}$min^{-1} (SE), PC= 1.45%<br>LFHCC n-3=2.53±0.18 (mul^{-1})$^{-1}$min^{-1} (SE), PC= -1.94%<br>HSFA=2.89±0.18 (mul^{-1})$^{-1}$min^{-1} (SE), PC= -6.47%					HMUFA= 9.86±0.51 ulU ml^{-1} (SE),<br>LFHCC= 10.31±0.52 ulU ml^{-1} (SE),<br>LFHCC n-3= 10.22±0.55 ulU ml^{-1} (SE),<br>HSFA= 9.83±0.54 ulU ml^{-1} (SE)	HMUFA= 9.20±0.56 ulU ml^{-1} (SE), PC= -6.6%<br>LFHCC= 10.22±0.57 ulU ml^{-1} (SE), PC= -0.87%<br>LFHCC n-3= 9.60±0.60 ulU ml^{-1} (SE), PC= -6.06%<br>HSFA= 10.05±0.59 ulU ml^{-1} (SE), PC= 2.23%
**Brady, 2004**	High n-6 group=2.04± 0.37 (μU min^{-1} mL^{-1} × 10^{-4}) (SE)	High n-6 group=1.73± 0.26 (μU min^{-1} mL^{-1} x 10^{-4}) (SE), PC= -15.2%			High n-6 group=2.24± 0.27 (SE)	High n-6 group=2.28± 0.28 (SE), PC= 17.9%		
**Fava, 2013**	HM/HGI (high monounsaturated fat/high glycaemic index) =3.49±2.85 (μU min^{-1} mL^{-1} x 10^{-4}) (SD),<br>HM/LGI (high monounsaturated fat/low glycaemic index) =3.78±2.17 (μU min^{-1} mL^{-1} x 10^{-4}) (SD),<br>HC/HGI (high carbohydrate/high glycaemic index)=2.75±1.55 (μU min^{-1} mL^{-1} x 10^{-4}) (SD),<br>HC/LGI (high carbohydrate/low glycaemic index)=2.86±1.1 (μU min^{-1} mL^{-1} x 10^{-4}) (SD),<br>HS (high saturated fat)=3.58±1.67 (μU min^{-1} mL^{-1} x 10^{-4}) (SD)	HM/HGI=3.02±1.28 (μU min^{-1} mL^{-1} x 10^{-4}) (SD), PC= -13.47%<br>HM/LGI=3.13±1.97 (μU min^{-1} mL^{-1} x 10^{-4}) (SD), PC= -17.2%<br>HC/HGI=2.93±1.48 (μU min^{-1} mL^{-1} x 10^{-4}) (SD), PC= 6.55%<br>HC/LGI=3.45±2.12 (μU min^{-1} mL^{-1} x 10^{-4}) (SD), PC= 20.63%<br>HS=3.21±1.38 (μU min^{-1} mL^{-1} x 10^{-4}) (SD), PC= -10.34%					HM/HGI=59.8±21.1 ϱmol l^{-1} (SD)<br>HM/LGI=67.8±25.2 ϱmol l^{-1} (SD)<br>HC/HGI=79.0±35.2 ϱmol l^{-1} (SD),<br>HC/LGI=73.3±37.7 ϱmol l^{-1} (SD),<br>HS=77.6±93.8 ϱmol l^{-1} (SD)	HM/HGI=61.5±25.4 ϱmol l^{-1} (SD), PC=2.84%<br>HM/LGI=68.1±32.1 ϱmol l^{-1} (SD), PC= 0.44%<br>HC/HGI=67.9±29.4 ϱmol l^{-1} (SD)**, PC= 14%<br>HC/LGI=70.6±35.4 ϱmol l^{-1} (SD), PC=3.68%<br>HS=72.8±80.4 ϱmol l^{-1} (SD), PC= -6.18%
**Giacco, 2013**	Whole grain=2.97±0.2(10^4 min^{-1}/(μU/ml)) (SE)	Whole grain= 3.05±0.19 (10^4 min^{-1}/(μU/ml)) (SE), PC= 2.69%					Whole grain=93.8±52.8 ϱmol l^{-1} (SD)	Whole grain= 103.5±55.6 ϱmol l^{-1} (SD), PC= 10.34%
**Juntunen, 2003**	Run in period=4.6±2 (10^{-4}min^{-1}X μU^{-1}XmL^{-1}) (SD)	Rye Bread (RB) period=4.1±2.1(10^{-4}min^{-1}X μU^{-1}XmL^{-1}) (SD), PC= -10.87%					Beginning of RB period=59.3±28.3 pmol/L (SD),	End of RB period=52.4±30.3 pmol/L (SD), PC= -11.63%<br>End of WB period= 49.0±20.3 pmol/L (SD), PC= -16.80%

**Kien, 2013**	Men HPA=5.48±0.77 (x10^{-4}mL μU^{-1} min^{-1}) (SE),<br>Women HPA=3.96±0.70 (x10^{-4}mL μU^{-1} min^{-1}) (SE)	Wheat Bread (WB)period=3.9±1.8 ($10^{-4}min^{-1}$X μU^{-1}XmL^{-1}) (SD), PC= -15.22%<br>Men HOA=5.31±1.05 (x10^{-4}mL μU^{-1} min^{-1}) (SE), PC= -3.1%<br>Women HOA=6.46±1.38 (x10^{-4}mL μU^{-1} min^{-1}) (SE)**, PC= 63.13%					Beginning of WB period=58.9± 25.8 pmol/L (SD)<br>Men Baseline=69±4.8 pmol/L (SE), Women baseline=73.8±4.1 pmol/L (SE)	Men HOA=66.4 ±4.9 pmol/L (SE), PC=-3.76%<br>Men HPA=61±6.1 pmol/L (SE), PC= -11.59%<br>Women HPA=91.5±21.8 pmol/L (SE), PC= 23.98%<br>Women HOA=65±5 pmol/L (SE)**, PC= 11.92%
**Douglas, 2006**	Baseline=1.9±1.7 (SD)	MUFA= 1.9±1.6 (x$10^{-4}min^{-1}$/ulU/mL) (SD), PC= 0%<br>LOW CHO=2.1±1.9 (x$10^{-4}min^{-1}$/ulU/mL) (SD), PC= 10.53%<br>STD=1.9±1.7 (x$10^{-4}min^{-1}$/ulU/mL) (SD), PC= 0%					Baseline=20.6±7.8 μIU/mL (SD)	MUFA=17.8±7.5 μIU/mL (SD), PC= -13.59%<br>LOW CHO=14.3± 8.2 μIU/mL (SD)**, PC= -30.58%<br>STD=17.5±7.2 μIU/mL (SD), PC= -15.04%
**Paniagua, 2007**		MUFA=3.9±0.6[x $10^{-4}min^{-1}$/(μU/ml)] (SE)**, PC=<br>CHO=3.5±0.4[x $10^{-4}min^{-1}$/(μU/ml)] (SE), PC = 11.42%<br>SAT=3.3±0.5[x $10^{-4}min^{-1}$/(μU/ml)] (SE), PC= -15.38%						MUFA=60.7±12.4 pmol/l (SE)<br>CHO=75.2±12.3 pmol/l (SE)**, PC= -19.28%<br>SAT=64.1±9.6 pmol/l (SE), PC= -14.76%
**Davis, 2012**	Nutrition=1.4±0.9 ($10^{-4}min^{-1}$μU mL^{-1}) (SD)	Nutrition= 1.6±0.2 ($10^{-4}min^{-1}$μU mL^{-1}) (SD)**, PC= 14.29%					Nutrition= 19.1±9.5 μU mL^{-1} (SD)	Nutrition= 16.1±2.0 μU mL^{-1} (SD)**, PC= -15.7%
**Davis, 2009**	Nutrition (N)= 1.6±0.9(10^{-4}/min/μU/ml) (SD),<br>Nutrition + strength training (NS)= 1.5±0.9(10^{-4}/min/μU/ml) (SD)	N=1.8±0.8(10^{-4}/min/μU/ml) (SD), PC= 12.5%<br>NS = 1.5±0.8(10^{-4}/min/μU/ml) (SD), PC= 0%	N= 459.3±319.5 nmol/min/l (SD),<br>NS= 405.0±279.1 nmol/min/l (SD)	N= 368.3±386.6 nmol/min/l (SD), PC= -19.81%<br>NS= 302.6 ±159.8 nmol/min/l (SD), PC= -25.28%	N= 6.1±4.2 (SD),<br>NS= 7.1±2.5 (SD),	N= 5.5±3.3 (SD), PC= -9.84%<br>NS= 6.1±3.0 (SD), PC= -14.08		

**p≤0.05, SD= Standard deviation, SE= Standard error of the mean. PC= Proportional change between baseline and intervention %, IVGTT (S_I)= Intravenous Glucose Tolerance Test, HOMA-IR=Homeostatic Model Assessment for Insulin Resistance, AUC OGTT= Area Under the Curve-Oral Glucose Tolerance Test.
