# Supplementary material for: The Use and Effectiveness of Selected Alternative Markers for Insulin Sensitivity and Secretion Compared with Gold Standard Markers in Dietary Intervention Studies in Individuals without Diabetes: Results of a Systematic Review
Source: Nutrients. 2022 May 12;14(10):2036. doi: 10.3390/nu14102036 (PMC9143618; doi:10.3390/nu14102036)
Supplement: Supplementary file 1 [file nutrients-14-02036-s001.zip › Supplemental Material Table S3.pdf]

Table S3. Results of the AIRg trials and DI (surrogate marker).

	AIRg		DI	
	**Baseline**	**End of intervention**	**Baseline**	**End of intervention**
**Osterberg, 2015**	VSL#3 probiotic= 481±102 [(mU L^{-1}) 10 min^{-1}] (SE)	VSL#3 probiotic= 523±90[(mU L^{-1}) 10 min^{-1}] (SE), PC= 8.73%	VSL#3 probiotic=2160±293 ($x10^{-4}$/min) (SE)	VSL#3 probiotic=2718±471 ($x10^{-4}$/min) (SE), PC= 25.83%
**Giacco, 2013**	Whole grain=61.4±8.4 (μU/ml) (SE)	Whole grain= 60.0±7.8 (μU/ml) (SE), PC= -2.28%	Whole grain= 140±17($x10^{-4}$/min) (SE)	Whole grain=144±17($x10^{-4}$/min) (SE), PC= 2.85%
**Kien, 2013**	Men HPA= 227.9±36.7 (min/mU/mL) (SE),	Men HOA= 250.3 ±41.6 (min/mU/mL) (SE), PC= 9.83%	Men HPA=1089.1±139.7 ($x10^{-4}$/min) (SE)	Men HOA=1071.1±143.8 ($x10^{-4}$/min) (SE), PC= -1.65%
	Women HPA=330.8±53.4 (min/mU/mL) (SE)	Women HOA= 325.5±68.3(min/mU/mL) (SE), PC= 1.6%	Women HPA=1137±198 ($x10^{-4}$/min) (SE)**	Women HOA= 1661±324($x10^{-4}$/min) (SE), PC= 46.08%
**Douglas, 2006**	Baseline=1234.7±1036.4 (uIU/mLx10 minutes) (SD)	MUFA= 1325.8±890.6(uIU/mLx10 minutes) (SD)**, PC=7.38%	Baseline=1406.4±869.8 ($x10^{-4}$/min) (SD)	MUFA= 1544.2±858.1 ($x10^{-4}$/min) (SD), PC= 9.81%
		LOW CHO= 1045.6±876.8(uIU/mLx10 minutes) (SD)**, PC= -15.32%		LOW CHO= 1516.4±1987.5 ($x10^{-4}$/min) (SD), PC= 7.82%
		STD= 1096.4±781.3(uIU/mLx10 minutes) (SD), PC= -11.2%		STD= 1412.3±939.4 ($x10^{-4}$/min) (SD), PC= 0.42%
**Davis, 2009**	N= 1160.4±869.9 μU $mL^{-1} \times$ 10 min (SD),	N= 1222.8±994.2 μU $mL^{-1} \times$ 10 min (SD), PC= 5.38%	N= 1297.1±596.7 ($x10^{-4}$/min) (SD)	N= 1723±898.3 ($x10^{-4}$/min) (SD), PC= 32.84
	NS= 1530.0±870.4 μU $mL^{-1} \times$ 10 min (SD)	NS= 1607.0±959.2 μU $mL^{-1} \times$ 10 min (SD), PC= 5.03%	NS= 1832.6±808.8 ($x10^{-4}$/min) (SD)	NS= 2002.9±994.3 ($x10^{-4}$/min) (SD), PC=9.27%

**$p \leq 0.05$. SD= Standard deviation, SE= Standard error of the mean, PC= Proportional change between baseline and intervention %, AIRg= Acute Insulin Response to glucose, DI= disposition index.
